# 

**Published:** 1845-05

**Authors:** 


### WESTERN JOURNAL

OF

### MEDICINE AND SURGERY.

### EDITORS.

---

DANIEL DRAKE, M. D.,

PROFESSOR OF PATHOLOGY AND THE PRACTICE OF MEDICINE
IN THE MEDICAL INSTITUTE OF LOUISVILLE:

LUNSFORD P. YANDELL, M. D.,

PROFESSOR OF CHEMISTRY AND PHARMACY IN THE SAME:

THOMAS W. COLESCOTT, M. D.

### BOOKS RECEIVED.

In future numbers, it is our expectation to notice at length several of the following works, but at present we are restricted to the briefest remarks on their merits.

*A Treatise on the Diseases and Special Hygiene of Females,* by Colombat De L'Isere, translated from the French by Charles D. Meigs, M.D. &c. We have received a copy of this elaborate work from the publishers, Messers. Lea & Blanchard. We hope in our next number to be able to present our readers with a review of it from the pen of one of our most valued correspondents. We have called the work an elaborate one, and the fact that references in it are made to "more than a thousand authorities" justifies that character.

*A Practical Treatise on the Diseases peculiar to women, illustrated by cases derived from Hospital and Private Practice.* By Samuel Ashwell, M. D. &c, with notes by Paul B. Goddard, M. D. &c. To the same spirited publishers we are indebted for this work, treating of subjects almost identical with those of the volume just above. The publication of two such volumes at the same time, from authors of different countries, is proof of the interest awakened in the profession by the complaints of females. We indulge the hope that the diffusion of so much light as these works from the pens of distinguished practitioners ought to impart, will be beneficially felt by our brethren, and by this interesting class of their patients.

*The Principles and Practice of Dental Surgery,* by Chapin Harris, M. D. &c. This is a second edition of Dr. Harris' compendious work on Dental Surgery. We are glad to see that the practitioners of that branch of the art appreciate a work which reflects so much credit upon their profession. We received our copy from Messrs. Lindsay & Blakiston, the publishers at Philadelphia. We shall have more to say about it at an early day; but mean time we advise every dentist to avail himself of the earliest opportunity of supplying himself with a copy of it.

*Principles and Illustrations of Pathological Anatomy,* by J. Hope, M. D. &c., edited by L. M. Lawson, M. D. &c. We are indebted for a copy of this admirable work to the politeness of the editor. The publishers have given it to the readers on a very large type and fair paper, making a volume of imposing size and show. Y.

### *Receipts of Medical Journal for April* 1845.

| | |
|---|---|
| Dr. W. Dempsy, Bremen, Ky., - - - - - | $5 00 |
| Dr. W. Miller, Hitesville, Ill., - . - - - - | 10 50 |
| Dr. S. Thompson, Albion, Ill., - - - - - - - | 5 00 |
| Dr. A. M. Ayres, Salem, Miss., - - - - - | 5 00 |
| Dr. M. B. Logan, Springfield, Ky., - - - - - | 15 00 |
| Dr. T. A. Ferris, Lawrenceville, Ark., - - - - | 5 00 |
| Dr. M. Mills, Youngstown, N. Y., - - - - - | 5 00 |
| Dr. H. P. Griswold, Plymouth, Ill., (postage 37) - - | 10 00 |
| Dr. J. Brooks, Egg's Point, Miss., - - - - - | 5 00 |
| Drs. Souell & Macklin, Athens, Ala., - - - - - | 5 00 |
| Dr. R. Searcy, Tuscaloosa, Ala., - - - - - | 5 00 |
| Dr. W. W. Higgins, Jones' P. O., Ill.,- - - - - | 16 25 |
| Dr. W. M. Bolling, Montgomery, Ala., - - - - | 5 00 |
| Dr. F. H. Hamilton, Buffalo, N. Y., - - - - - - | 1 00 |
| Dr. J. Mitchell, Richmond, La., - - - - - - - | 10 00 |
| Dr. M. Shepherd, Paysou, Ill., - - - - - - - | 5 00 |
| Dr. D. J. Edwards, Leavenworth, Ind., - - - - | 5 00 |

### TO SUBSCRIBERS.

We regret to find that, notwithstanding unusual expenditures upon the present volume of this Journal and its very material improvement, our subscribers have this year been more than usually remiss. We would give notice that all who may not remit promptly shall be forthwith and constantly harrassed with every form of dun until they are worried out. Their names shall appear in a black list on the back of the Journal, and, simultaneously, they shall be called upon and persecuted by duns of every kind, travelling agents, local agents, constables, sheriffs, and catchpoles. We are shamefully deprived of our dues and we mean to have justice done us.

PRENTICE & WEISSINGER.

☞Receipt by mail, at our risk, under frank of post-master or post-paid.

---

### TRAVELING AGENTS FOR THE WESTERN JOURNAL OF MEDICINE AND SURGERY.

JNO. F. JOHNSTON and JAS. H. STEVENSON, Kentucky—W. H. & W. M. WHEELER, Georgia, N. Carolina, S. Carolina, Alabama, and Arkansas—C. W. JAMES, Ohio, Indiana, and Michigan—GEO. C. BRETT, Tennessee—J. F. EASTON, Illinois and Missouri—WM. H. WELD & SON, Mississippi and Louisiana.

---

The WESTERN JOURNAL OF MEDICINE AND SURGERY is published monthly by the undersigned, at the corner of Main and Fifth streets, Louisville, at $5 per annum, payable in advance. Each number contains 96 pages, making two volumes in the year of more than 550 pages.

Contributions to its pages by the Physicians of the Valley of the Mississippi, addressed to the Editors, are respectfully solicited.

Letters on business, to be addressed, postage paid, to the publishers.

☞Postmasters, by the regulations of the Postoffice Department, will frank letters containing subscription money, and all remittances so franked are at the risk of the publishers.

*January*, 1844. PRENTICE & WEISSINGER.

